# Comparative analysis of scalp and gut microbiome in androgenetic alopecia: A Korean cross-sectional study

**DOI:** 10.3389/fmicb.2022.1076242

**Published:** 2022-12-12

**Authors:** Da-Ryung Jung, Hye-Young Yoo, Min-Ji Kim, Vineet Singh, Sung-Ha Park, Minsoo Jeong, Byoung-Jun Park, Jae-Ho Shin

**Affiliations:** ^1^Department of Applied Biosciences, Kyungpook National University, Daegu, South Korea; ^2^Skin and Natural Products Lab, Kolmar Korea Co., Ltd., Seoul, South Korea; ^3^Department of Integrative Biotechnology, Kyungpook National University, Daegu, South Korea; ^4^NGS Core Facility, Kyungpook National University, Daegu, South Korea

**Keywords:** androgenetic alopecia, scalp microbiome, gut microbiome, amplicon sequencing, network

## Abstract

Androgenetic alopecia (AGA) is a non-scarring and progressive form of hair loss occurring in both men and women. Although genetic predisposition and sex steroid hormones are the main causes, many factors remain unknown, and various extrinsic factors can negatively affect the lifespan of hair. We investigated skin–gut axis microorganisms as potential exogenous factors causing AGA, through comparative analyses of the scalp and gut microbiome in individuals with and without AGA in a Korean cohort. Using 16S rRNA gene sequencing, we characterized the scalp and gut microbiomes of 141 individuals divided into groups by sex and presence of AGA. Alpha diversity indices in the scalp microbiome were generally higher in individuals with AGA than in healthy controls. These indices showed a strong negative correlation with scalp-inhabitant bacteria (*Cutibacterium* and *Staphylococcus*), indicating that the appearance of non-inhabitant bacteria increases as hair loss progresses. No significant differences in diversity were observed between the gut microbiomes. However, bacterial functional differences, such as bile acid synthesis and bacterial invasion of epithelial cells, which are related to intestinal homeostasis, were observed. The networks of the scalp and gut microbiome were more complex and denser with higher values of the network topology statistic coefficient values (i.e., transitivity, density, and degree centrality) and more unique associations in individuals with AGA than in healthy controls. Our findings reveal a link between skin–gut microorganisms and AGA, indicating the former’s potential involvement in the latter’s development. Additionally, these results provide evidence for the development of cosmetics and therapeutics using microorganisms and metabolites involved in AGA.

## Introduction

Alopecia is a generic term for a comprehensive form of hair loss in both men and women, including androgenetic alopecia (AGA), alopecia areata (AA), and cicatricial alopecia (Tosti and Duque-Estrada, 2009). AGA is the most common case of progressive hair loss, in which hair miniaturization occurs as the anagen phase shortens and telogen is prolonged during hair growth cycles on the parietal area and vertex of the scalp (Trüeb, 2002). Treatment for alopecia is time-consuming and difficult; people with hair loss have a lower quality of life, such as lower self-confidence and higher feelings of depression (Lee et al., 2002; Yeo et al., 2014; Marks et al., 2019). Therefore, the management of hair loss can be an important part of people’s lives.

Genetic predisposition and steroid sex hormones are important factors in the development of AGA (Yip et al., 2011; Vary, 2015). Studies in identical twins by Gatherwright et al. provided evidence that, in addition to heredity, exogenous factors had clinically significant effects on male and female AGA (Gatherwright et al., 2012, 2013). Later studies confirmed that various exogenous factors including metabolism, psychological changes, environmental exposure, dietary intake, and microorganisms can negatively affect the lifespan of hair (Lai et al., 2013; Yeo et al., 2014; Phillips et al., 2017; Ho et al., 2019; Suzuki et al., 2021). Therefore, we investigated microorganisms as potential exogenous factors of AGA.

The skin is a complex and dynamic ecological system inhabited by commensal microbes and opportunistic pathogens that interact with their host (Chen et al., 2018). Skin microorganisms contribute to host immunity and inflammatory activity; therefore, they could be related to hair diseases, such as hair loss and miniaturization (Dréno et al., 2016; Ho et al., 2019). Suzuki et al. reported that *Cutibacterium*, *Corynebacterium*, and *Staphylococcus* are the most abundant genera on the scalp surface, and scalp dysbiosis occurs in patients with AGA (Suzuki et al., 2021). The scalp condition of patients with AA was associated with more abundant *Cutibacterium acne* and fewer *Staphylococcus epidermis* compared to that of healthy controls (Pinto et al., 2019). Additionally, *C. acnes* is associated with immune response gene expression in hair follicles and can be involved in AGA pathogenesis and hair miniaturization (Ho et al., 2019). Other scalp disorders, such as dandruff and seborrheic dermatitis, are also associated with the relative abundance of *Cutibacterium* spp. and *Staphylococcus aureus* (Park et al., 2017).

The gut microbiome functions as a regulator of the skin–gut axis by modulating cutaneous immune response and chronic inflammatory conditions (Salem et al., 2018). Conteville et al. showed that the gut microbiome is affected by exposure of the skin to ultraviolet type-B light radiation, indicating an interrelationship between the microbiomes (Conteville and Vicente, 2020). An association between the gut microbiome and AA has been suggested too (Moreno-Arrones et al., 2020). Cases of hair regrowth after fecal microbiota transplantation in patients with AA have been reported (Rebello et al., 2017). Furthermore, intestinal microbiome imbalance, especially the overgrowth of *Lactobacillus murins*, lowers intestinal metabolic function, leading to alopecia (Hayashi et al., 2017).

There have been multiple microbiome studies on the skin–gut axis, alopecia, and scalp diseases; however, simultaneous analysis of the scalp and gut microbiome in AGA has not been conducted, and comparative studies between men and women are lacking. Therefore, we investigated the differences in the scalp and gut microbiome in individuals with and without AGA in a Korean cohort and studied the microbial communities by subdividing the groups according to the degree of symptom severity and sex. Our study suggests a new microbiological perspective of AGA based on the scalp and gut microbiome analysis results.

## Materials and methods

### Study design and microbial sample collection

**Figure 1:**
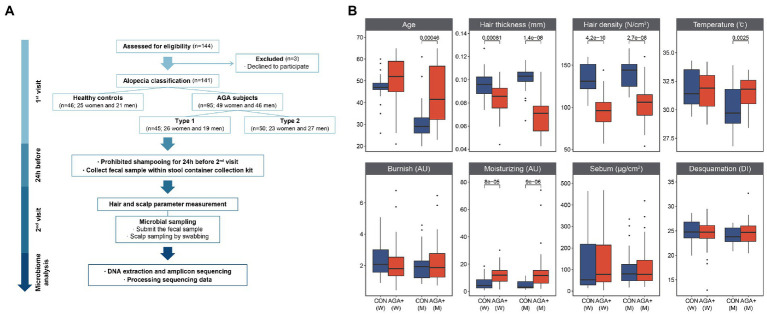
Study design and comparison of hair and scalp parameters between healthy controls (CON) and individuals with AGA (AGA+). **(A)** Flow of the study design. **(B)** Differences between CON and AGA+ with respect to hair and scalp parameters. W and M are abbreviations for women and men, respectively.

A total of 141 Korean women and men participated in this study which was conducted from June 2020 to June 2021 at Kolmar Korea Co. (Seoul, South Korea). This study was approved by the Korean Public Institutional Review Board (IRB) of the Korea National Institute for Bioethics Policy (IRB: P01-202002–33-001). A flow diagram of the procedures and conditions is shown in Figure 1A. The participants were divided into two groups according to the presence or absence of hair loss by referring to the basic type (BA) and specific type (SP) classification criteria (BASP) through visual assessment by the researchers: (1) 46 healthy controls (CON, 25 women and 21 men) and (2) 95 individuals with AGA (AGA+, 49 women and 46 men). Based on the severity of hair loss, the AGA+ group was further divided into two groups (type-1 and type-2). Type-1 refers to a specific type (V1 or F1) of slightly wider hair parting than normal (L, M0, C0); type-2 refers ta o specific type (V2-3 or F2-3) of hair parting wider than that occurring during type. Participants who met the following criteria were excluded: (1) have undergone a surgical procedure for alopecia or any infectious disease on the scalp, (2) have taken probiotics in the last 15 days, and (3) have been administered any antibiotics within 1 month. Before sampling, the use of hair care products and shampoos was prohibited for 1 day. Sampling and clinical evaluation were conducted in a room maintained at constant temperature (24 ± 2°C) and humidity (50 ± 5%). Scalp microorganisms were collected using a sterilized cotton tip and previously described preservatives (Kim et al., 2021b). The parietal scalp and vertex (15–25 cm^2^) (Supplementary Figure 1) were swabbed for 3 min. To collect gut microbial samples, the participants were asked to provide fresh fecal samples. All microbial samples were immediately stored at −80°C until DNA extraction.

### Hair and scalp clinical assessment

Seven clinical hair (thickness, density, and gloss) and scalp (moisturizing, sebum, desquamation, and temperature) parameters were measured at the scalp microorganism collection site. The parameters were evaluated using the following methods: (1) the USB-based hand-held imaging device Folliscope® (Coscam CCL- 215 USB, Sometech, South Korea) was used to measure hair thickness and density. Images of the scalp site containing hair were captured and analyzed using Folliscope 2.8 software. Hair thickness was calculated by taking the average of three randomly selected hair strands; density was determined by measuring the number of hair strands present in the pores within the range of the image. (2) Glossymeter® GL200 (Courage & Khazaka, Cologne, Germany) was used to obtain the gloss diffusion scattering correction value by measuring the gloss based on the light reflected from the hair surface 10 consecutive times. (3) Corneometer® CM825 (Courage & Khazaka) was used to obtain the moisture content of the scalp (stratum corneum—the outermost layer of the skin) by measuring electrical capacitance five times. (4) Sebumeter® SM815 (Courage & Khazaka) was used to obtain the casual sebum level of the scalp by applying a constant pressure for 30 s to measure the sebum after calibrating to zero with an unused tape. (5) Corneofix® and Visioscan ® VC98 (Courage & Khazaka) were used to obtain the dead cell level of the scalp. After attaching Corneofix (a transparent adhesive tape) to the scalp, it was pressed with the thumb at the same pressure for 5 s to collect corneocytes, and the collected corneocytes were visualized and the desquamation index was analyzed using Visioscan. (6) A thermometer was used to measure the temperature of the parietal scalp.

### DNA extraction and amplicon sequencing

Scalp and gut bacterial DNA were extracted using a modified method to increase the DNA extraction efficiency with the DNeasy PowerSoil Pro Kit and DNeasy PowerFecal Pro Kit (Qiagen, Hilden, Germany), respectively. The quantity and quality of the extracted DNA were confirmed using a Qubit® 2.0 Fluorometer (Life Technologies, Carlsbad, CA, United States) and NanoDrop 2000/2000c Spectrophotometer (Thermo Fisher Scientific, Waltham, MA, United States), respectively. We gave priority to the detection of various bacteria rather than distinguish species of specific genus. Therefore, referring to study of (Walters et al., 2016), modified primers of the V4-V5 region of the 16S rRNA gene were designed to improve coverage when assigning bacterial taxonomic information using SILVA database. The sequences of the V4-V5 primer we designed are as follows: 16S-515F (5′-barcode-CGCTCTTCCGATCTGTGNCAGCMGCCGCGGTRI-3′) and 16S-907R (5′-barcode-GTGCTCTTCCGATCCGYCWATTYHTTTRAGTTT-3′). Finally, all libraries were multiplexed in equimolar amounts and paired-end (2 × 300 bp) sequenced using the Illumina MiSeq platform (Illumina, San Diego, CA, USA) at the KNU NGS core facility (Daegu, South Korea).

### Sequencing data processing

Raw sequence data were analyzed using the Quantitative Insights into Microbial Ecology 2 (QIIME2) pipeline (version 2021.4). In the raw amplicon data, the mean frequency was 35,272 reads for scalp microbiome and 29,834 reads for gut microbiome. To identify amplicon sequence variants (ASVs), the sequences were demultiplexed, trimmed, and denoised with DADA2 (Q score > 30, uniform length of 240 bp). Sequences of ASVs were taxonomically identified *via* the scikit-learn naive Bayes machine-learning classifier using the Silva v138 database, with a threshold of 99% sequence identity (silva-138-99-nb-classifier.qza). Mitochondrial, chloroplast, and unassigned taxa sequences were removed. Sequences of the scalp and fecal bacterial samples were rarefied to sequencing depths of 3,756 and 8,062 reads, respectively, to normalize the different sequencing depths of each sample for further analysis. The rarefaction curves were attached in Supplementary Figure 2. The functional profiles of the bacterial microbiome were predicted using Phylogenetic Investigation of Communities by Reconstruction of Unobserved States (PICRUSt2) v.2.3.0-b software. We then inferred the Kyoto Encyclopedia of Genes and Genomes (KEGG) pathway from the predicted KEGG ortholog abundance.

### Statistical analysis

All statistical analyses and visualization of the results in this study were performed using R software (version 4.0.2.^1^) Alpha diversity indices (Chao, phylogenetic diversity, and Simpson’s index) of the scalp and gut microbiomes were calculated using the phyloseq (version 1.32.0) (McMurdie and Holmes, 2013) and metagMisc (version 0.0.4;^2^) R packages. The Wilcoxon rank-sum test was used to compare CON and AGA+. Correlation analysis was performed using Spearman’s rank correlation coefficient with the Hmisc (version 4.5–0) (Harrell and Harrell, 2019) and Corrplot (version 0.90) (Wei et al., 2017) R packages. Beta diversity was computed with Bray–Curtis dissimilarity and presented as a principal coordinates analysis (PCoA) plot using vegan (version 2.5–7) (Wei et al., 2017) R package. The distances between the samples within the groups were calculated using QIIME2. To evaluate the statistical significance of the taxonomic profile and bacterial functional pathways, the Wilcoxon rank-sum test was applied, and taxa and pathways were filtered at *p* < 0.1. Heat maps for functional profiling were created using the gplots (version 3.1.1) (Warnes et al., 2016) R package. Co-occurrence analysis of the network was performed using the igraph (version 1.2.6) (Csardi and Nepusz, 2006) R package.

## Results

### Clinical evaluation of hair and scalp

The mean age in each group was 47.48 ± 7.19 years for CON and 49.96 ± 11.37 years for AGA+ in women, and 32.05 ± 9.64 years for CON and 43.61 ± 12.98 years for AGA+ in men (Figure 1B; Supplementary Figure 3). The differences in average hair thickness and density of women and men were as follows: (1) thickness: CON, 0.095 ± 0.013 mm (women) and 0.100 ± 0.012 mm (men) vs. AGA+, 0.084 ± 0.012 mm (women) and 0.069 ± 0.015 mm (men); (2) density: CON, 133.80 ± 17.99 cm^2^ (women) and 142.00 ± 17.94 cm^2^ (men) vs. AGA+, 95.24 ± 17.36 cm^2^ (women) and 104.02 ± 20.99 cm^2^ (men). Scalp temperature was higher in AGA+ (31.57 ± 1.27°C) than in CON (30.11 ± 1.96°C) only in men. The hydration parameters showed significant differences in both women and men. For women, the average values were 6.08 ± 4.94 AU and 12.28 ± 6.70 AU in CON and AGA+, respectively; for men, the values were 4.50 ± 3.16 AU and 14.15 ± 13.88 AU in CON and AGA+, respectively.

### Diversity of scalp and gut microbiome

In the scalp and gut microbiome, the bacterial communities of women and men were clearly distinct (Supplementary Figure 4). Therefore, we decided to distinguish between women and men in the following scalp and gut microbiome analyses.

**Figure 2:**
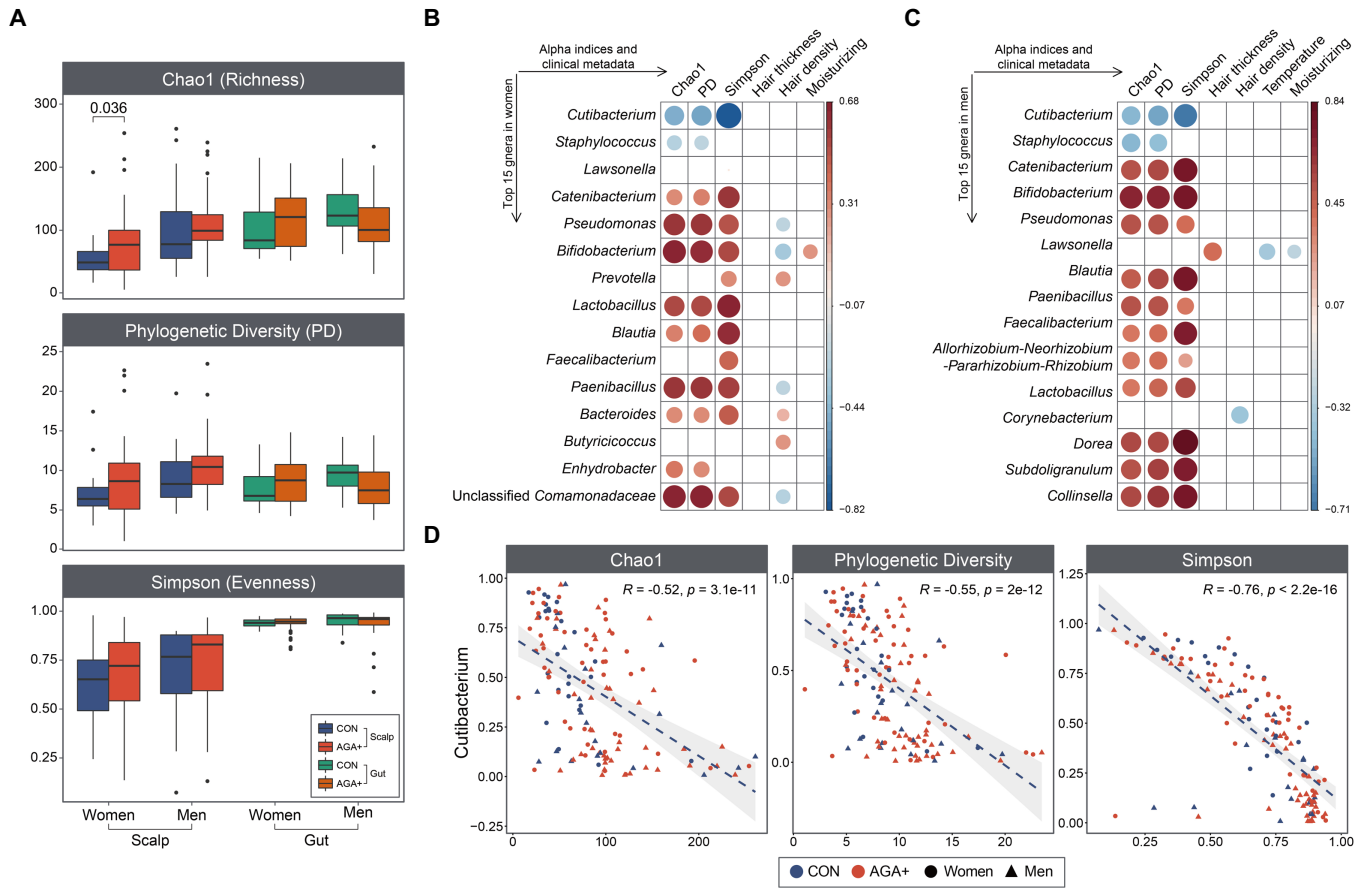
Alpha diversity of scalp and gut microbiome, and correlation pattern analysis. **(A)** The boxplots show the differences in the alpha diversity indices (Chao index, phylogenetic diversity, and Simpson’s index) between healthy controls (CON) and individuals with AGA (AGA+). Spearman’s correlation among the most abundant genera (women, **B**; men, **C**), alpha diversity indices, and clinical metadata. The color and size of the circles depict Spearman’s coefficient rho with a significant correlation (*p* < 0.05). **(D)** Scatter plots depicting the correlation between *Cutibacterium* and alpha diversity indices of scalp microbiome.

We compared the scalp and gut bacterial structures of CON and AGA+ using alpha diversity indices (Figure 2A). In the scalp microbiome, both women and men showed a higher tendency for alpha diversity indices in AGA+, but only the Chao index reached significance in women (*p* < 0.05, Wilcoxon rank-sum test). Even when the groups were divided according to AGA severity, a gradual increase in alpha diversity values was observed (Supplementary Figure 5). The gut microbiome did not show any significant differences in alpha diversity based on the presence or absence of AGA.

Correlation analysis of the top 15 most abundant genera with alpha diversity indices and clinical data showed strong correlations between genera and alpha diversity but weak correlations between genera and clinical data (Figures 2B,C). *Cutibacterium* and *Staphylococcus* were significantly (*p* < 0.05) negatively correlated with alpha diversity indices; most of the other taxa showed positive correlations with the indices. Notably, *Cutibacterium* showed significant negative correlations with all alpha diversity indices (Figure 2D). *Staphylococcus* was negatively correlated with Chao diversity and phylogenetic diversity (Supplementary Figure 6). Moreover, especially in women, hair density was positively correlated with *Prevotella*, *Bacteroides*, and *Butyricicoccus* but negatively correlated with *Pseudomonas*, *Bifidobacterium*, *Paenibacillus*, and Unclassified Comamonadaceae. In men, a significant negative correlation was observed between *Lawsonella* and temperature and moisture of the scalp.

**Figure 3:**
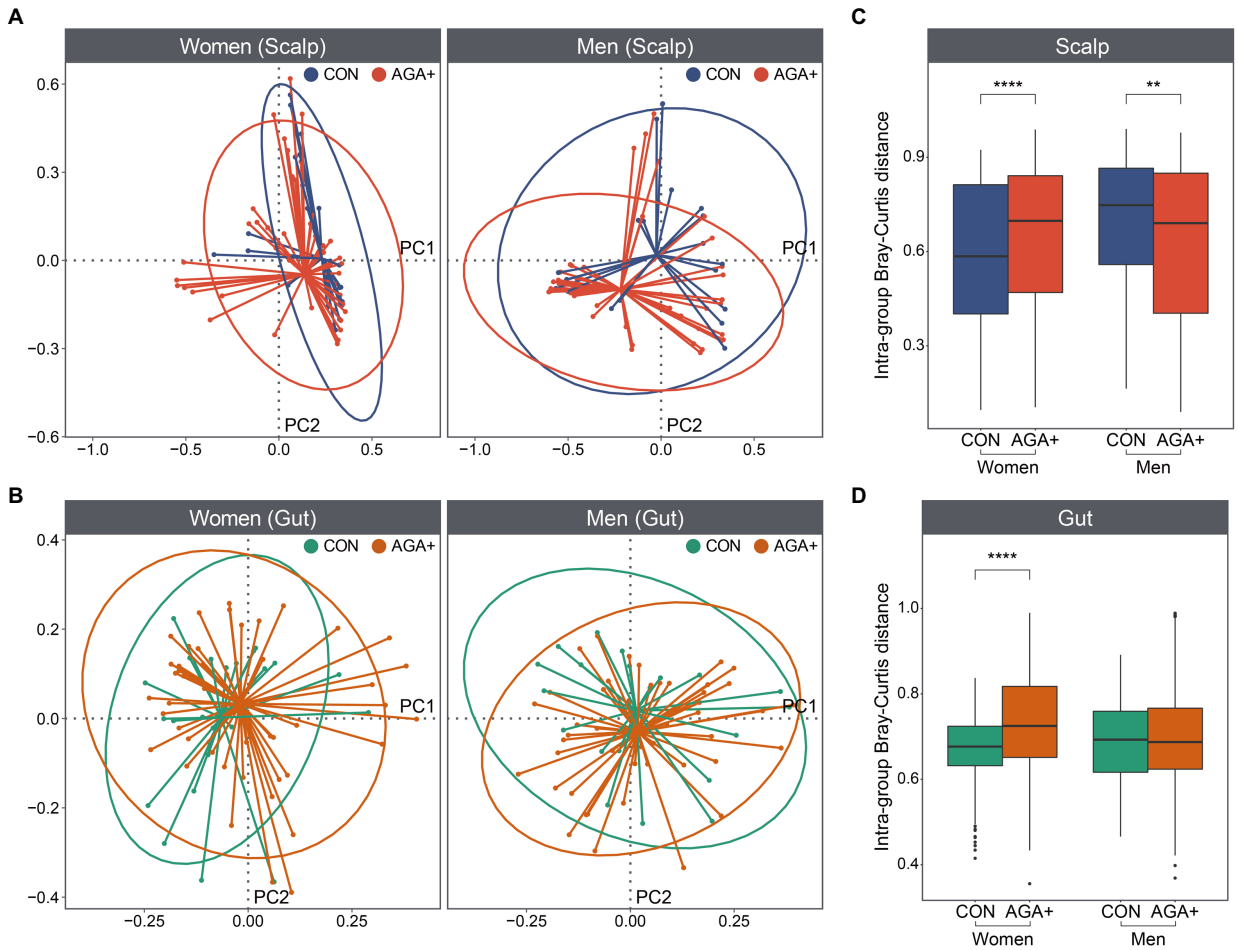
Beta diversity of scalp and gut microbiome. Principal coordinates analysis plot (based on Bray–Curtis distances) of the **(A)** scalp and **(B)** gut microbiome of healthy controls (CON) and individuals with AGA (AGA+). Ellipses represent a 95% confidence interval level for each group. The average Bray–Curtis distances between samples within each group in scalp **(C)** and gut **(D)** microbiome are shown in the boxplot.

We compared the scalp and gut microbiome structure of each group using PCoA based on Bray–Curtis dissimilarity (Figures 3A,B; Supplementary Figure 7). Although we did not observe a distinct separation between CON and AGA+, the distances between the samples within the groups were significantly different (Figures 3C,D). In the scalp microbiome, the intragroup distance was higher in AGA+ for women, but higher in CON for men. Additionally, in the gut microbiome, distance value in AGA+ was significantly higher than in CON, but only in women.

### Taxonomic profiling of scalp and gut microbiome

*Cutibacterium* and *Staphylococcus* predominated in scalp bacterial communities, followed by *Lawsonella* (women) and *Catenibacterium* (men) (Supplementary Table 1; Supplementary Figure 8A). The cumulative relative abundances of the three most abundant genera were 74.7% (CON) and 65.1% (AGA+) in women, and 57.3% (CON) and 53.7% (AGA+), respectively. In the gut microbiome, *Blautia* and *Faecalibacterium* were the most abundant in both women and men (Supplementary Table 2; Supplementary Figure 8B).

**Figure 4:**
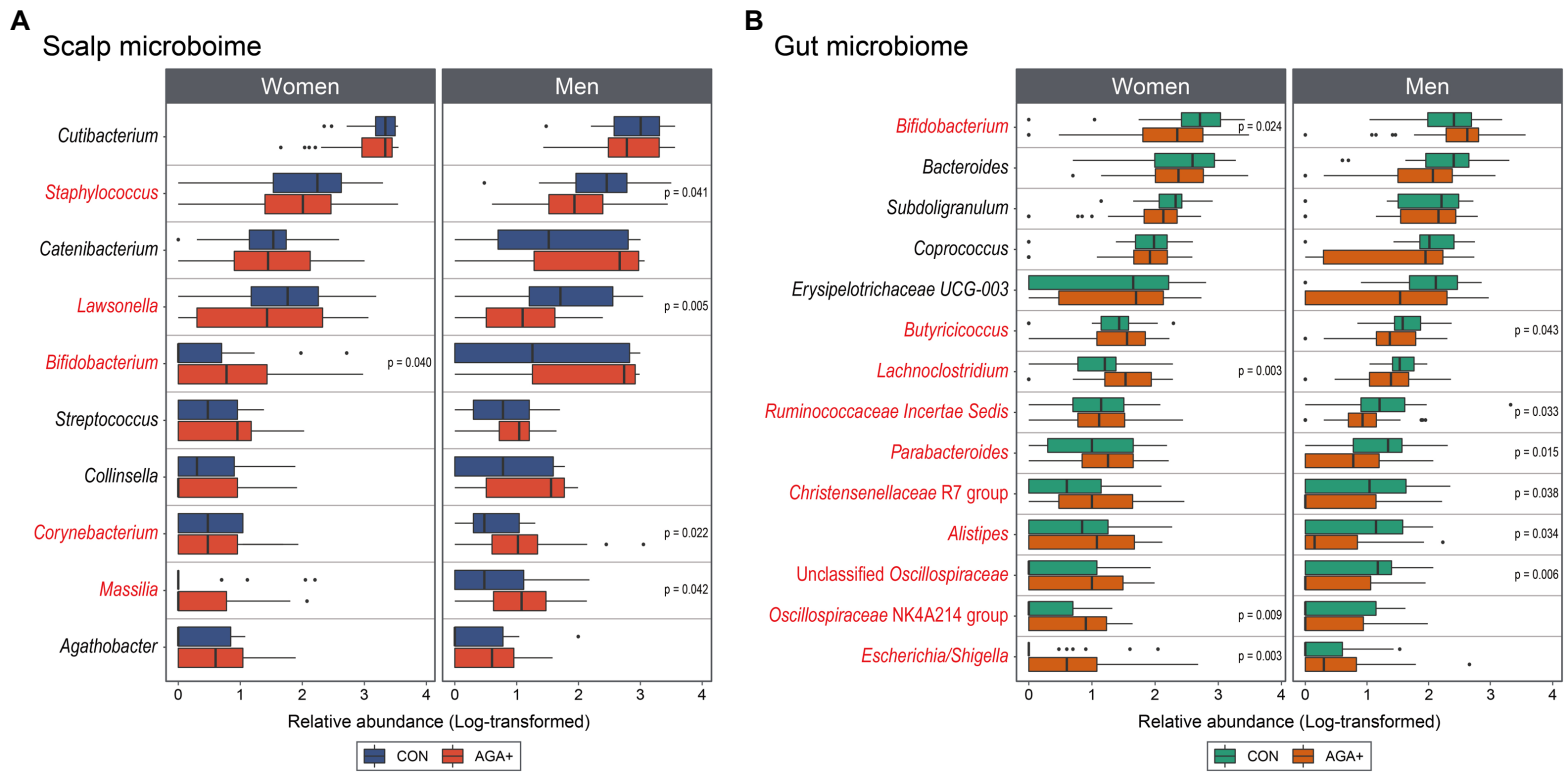
Differential abundance analysis of scalp **(A)** and gut **(B)** microbiome between healthy controls (CON) and individuals with AGA (AGA+) at the genus level. Boxplots represent log-transformed abundances of individual genera. Taxa with *p* < 0.05 or 0.01 determined using the Wilcoxon rank-sum test are indicated with asterisks (* and **, respectively). The genera marked in red are significantly different (CON vs. AGA+) in at least one women or men group.

Concerning the scalp microbiome between CON and AGA+, one and four taxa were found to be differentially abundant (*p* < 0.05, Wilcoxon rank-sum test) in women and men, respectively (Figure 4A). Looking at the most abundant genera on the scalp first, *Cutibacterium* showed no significant difference between CON and AGA+ in both women and men, but *Staphylococcus* was significantly less abundant in AGA+ only in men (*p* < 0.05, Wilcoxon rank-sum test). Furthermore, *Bifidobacterium* was significantly enriched in AGA+ in women (*p* < 0.05, Wilcoxon rank-sum test). In men, the abundance of three taxa, *Lawsonella*, *Corynebacterium*, and *Massilia*, was significantly different between CON and AGA+ (*p* < 0.05, Wilcoxon rank-sum test).

In the gut microbiome, women and men differed in four and ten taxa, respectively (Figure 4B). In women, the abundance of *Bifidobacterium*, *Lachnoclostridium*, Oscillospiraceae NK4A214 group, and *Escherichia*/*Shigella* was significantly different (*p* < 0.05, Wilcoxon rank-sum test). In men, *Butyricicoccus*, Ruminococcaceae incertae sedis, *Parabacteroides*, Christensenellaceae R7 group, *Alistipes*, and Unclassified Oscillospiraceae were significantly higher in CON than in AGA+ (*p* < 0.05, Wilcoxon rank-sum test).

### Bacterial functional profiling of the scalp and gut microbiome using PICRUSt2

**Figure 5:**
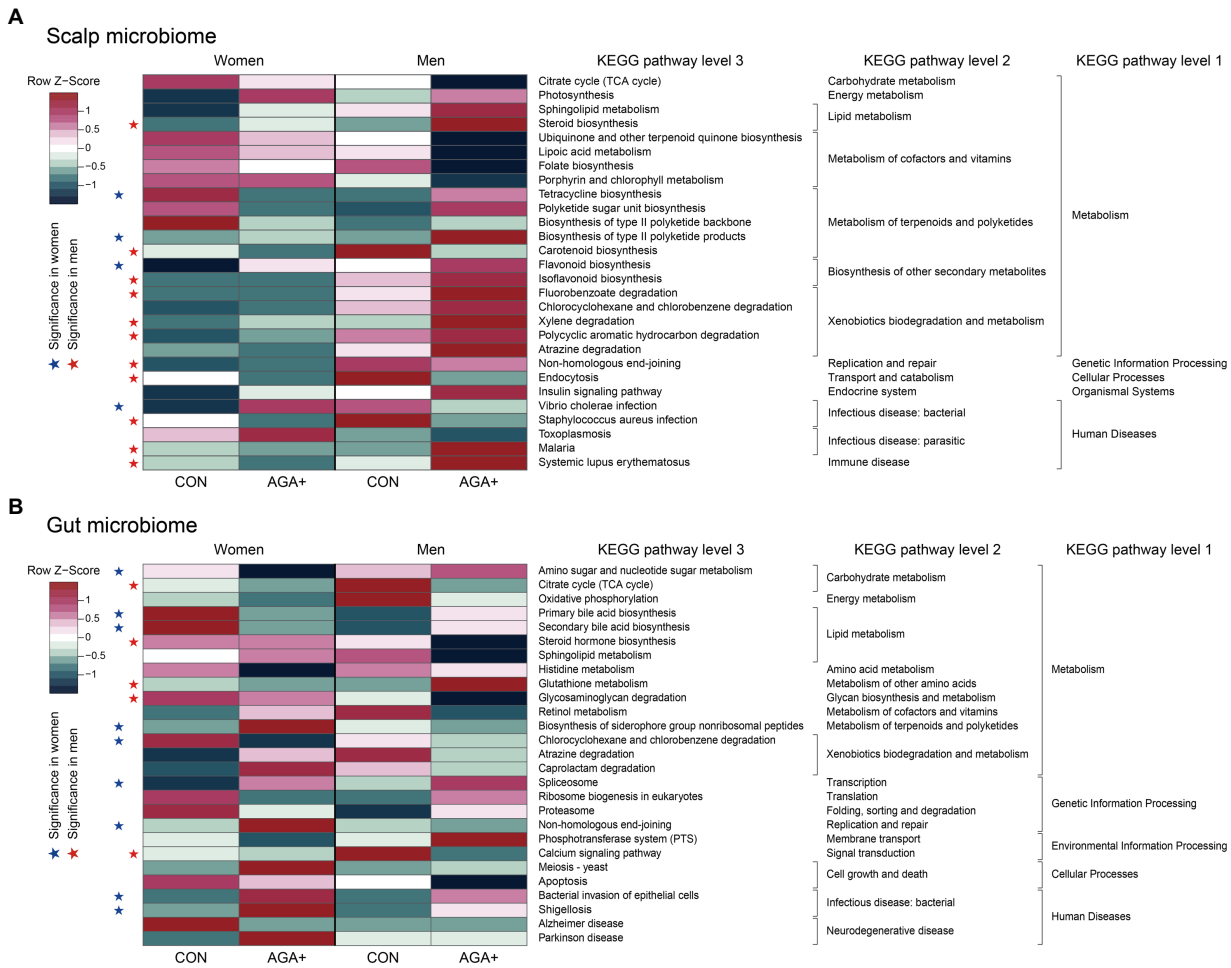
Heat map of differential bacterial functions predicted using PICRUSt2 in scalp **(A)** and gut **(B)** microbiome. The relative abundance of each microbiome was normalized based on row Z-scores. Blue (women) and red (men) stars indicate functions with significant differences between healthy controls (CON) and individuals with AGA (AGA+).

Based on the PICRUSt2 results, we defined 28 and 27 differentially abundant KEGG pathways between CON and AGA+ in the scalp and gut microbiome, respectively, (*p* < 0.1, Wilcoxon rank-sum test) (Figure 5). In the scalp microbiome, lipoic acid metabolism and folate biosynthesis pathways (in the metabolism of cofactors and vitamins category) were slightly more enriched in CON, while sphingolipid metabolism and steroid biosynthesis pathways (in the lipid metabolism category) were dominant in AGA+ (Figure 5A). In men, pathways belonging to the xenobiotics biodegradation and metabolism category were significantly abundant in AGA+. Concerning the gut microbiome of AGA+, bile acid biosynthesis pathways (primary and secondary bile acid biosynthesis) were predominant in women and steroid hormone biosynthesis was predominant in men (Figure 5B). Pathways involved in bacterial infectious diseases (bacterial invasion of epithelial cells and shigellosis) were more abundant in AGA+ than in CON.

### Scalp and gut bacterial community network

**Figure 6:**
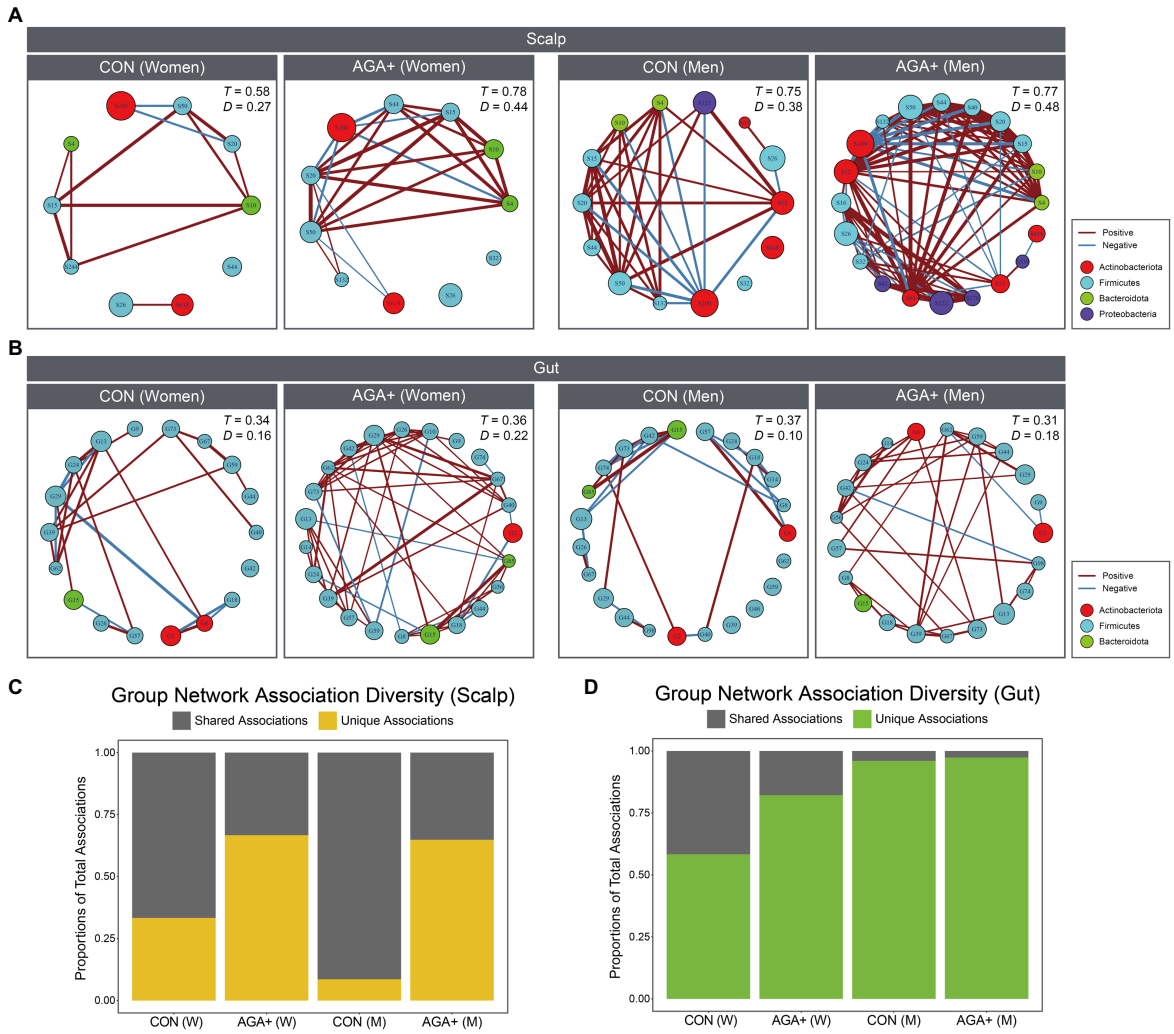
Bacterial association networks of scalp **(A)** and gut **(B)** microbiome at the genus level. Nodes indicate genera, and the size of each node represents the relative abundance. Edge color (red or blue) represents positive or negative correlations, respectively. The *T* and *D* values represent the transitivity and density of the network, respectively. The portion of associations within networks of each group that are unique or shared in scalp **(C)** and gut **(D)** microbiome. W and M are abbreviations for women and men, respectively.

To confirm differences in the bacterial community network between CON and AGA+, we performed co-occurrence analysis of the scalp and gut microbiomes (Figures 6A,B). We used genera that were relatively abundant and present in more than 70% (scalp) and 80% (gut) of samples in at least one group; detailed information on the taxa used is given in Supplementary Tables 3, 4. Using Spearman’s correlation, edges only represent *p* < 0.05, and their thickness indicate the strength of the correlation between nodes. Supplementary Table 5 presents the differences in the network interactions between groups, regarding transitivity, graph density, degree centralization, vertex number, and edge number. Network analysis showed that in the scalp microbiome, network density (D) was higher in AGA+ (women = 0.44; men = 0.48) than in CON (women = 0.27; men = 0.38). Similarly, in the gut microbiome, the D value was higher in AGA+ (women = 0.22, men = 0.18) than in CON (women = 0.16, men = 0.10). In the scalp and gut microbiomes, we observed a higher unique association in AGA+ than in CON (Figures 6C,D).

## Discussion

AGA is a non-scarring and progressive form of hair loss occurring in both men and women (Tamashunas and Bergfeld, 2021); however, the factors and patterns are slightly different between sexes. Although systemic androgens and genetic factors are the main causes of AGA (Salman et al., 2017) various exogenous factors also contribute to its development (Choi et al., 2017; Davis and Callender, 2018). Therefore, we compared the scalp and gut microbiomes of CON and AGA+ by sex and severity of AGA. Additionally, the clinical parameters of the hair and scalp were measured, and their association with the microbiome was investigated.

As expected, both hair thickness and density were significantly lower in AGA+ than in CON. Interestingly, the moisture content of the scalp was higher in AGA+ in both women and men. Harries et al. (2015) reported increased scalp sweating in women with frontal fibrosing alopecia, suggesting a possible association between increased sweating and scalp inflammation. Although recent studies (Chanprapaph et al., 2021; Suzuki et al., 2021) have suggested that AGA can be affected by changes in sebum composition, we found no differences in sebum in our results.

Similar to the results of Suzuki et al. (2021) and Pinto et al. (2019), in the scalp microbiome of our cohort, the alpha diversity of AGA+ showed an increasing trend compared with that of CON, especially for the Chao indices (bacterial richness) in women. The high Chao index of AGA+ could be derived to the significant increase in the number of non-skin inhabitant bacteria. However, in the study of Suzuki et al., all age groups were analyzed separately because age is closely related to development of AGA, but we did not analyze separately by dividing the age groups. This is because when we divided the groups according to age (e.g., 20s, 30s, 40s, 50s, and 60s), the trends in the results of alpha diversity were similar to those when all groups were combined (Supplementary Figure 9). Also, a higher diversity of the scalp microbiome was found in older adults than in young adults in a Japanese women cohort (Shibagaki et al., 2017). The authors noted that this result was due to an increase in the minor operational taxonomic unit/species, which may reflect overall structural changes. In addition, that moist skin regions create favorable conditions for microorganisms and are colonized by various species (Cundell, 2018; Skowron et al., 2021). It may support the increased microbial diversity in AGA+ with a high moisture content of the scalp.

At the genus level, the scalp microbiome of the participants mostly consisted of *Cutibacterium* and *Staphylococcus*. Our results support the findings of previous studies that these 2 g-positive bacterial genera are most dominant on the scalp surface (Saxena et al., 2018; Grimshaw et al., 2019). *Cutibacterium* and *Staphylococcus* are the most abundant bacterial genera on skin, and they maintain skin health by regulating the immune response (Fournière et al., 2020; Nakatsuji et al., 2021). In particular, *Cutibacterium* maintains skin homeostasis and plays a role in lipid modulation, follicular niche competition, immune regulation, and oxidative stress mitigation (Rozas et al., 2021). Based on our results, we inferred that the decline in the abundance of these two bacterial genera leads to an imbalance between skin inhabitant and non-inhabitant bacteria. Our results were contradicted with the results of Suzuki et al. (2021) that *Corynebacterium* decreased and *Staphylococcus* increased in the AGA group, but were consistent with the results of increased *Corynebacterium* and decreased *Staphylococcus* (Pinto et al., 2019; Won et al., 2022) in the AA group. In particular, Won et al. noted that *Corynebacterium* and *Staphylococcus* spp. could be used to predict a worse prognosis of AA. Also, in our cohort, *Corynebacterium* was strongly negatively correlated with hair density in men, and this genus was found to be associated with skin aging on the forehead (Dimitriu et al., 2019). *Lawsonella*, which was negatively correlated with moisture, was observed in a recent skin microbiome study in Korea (Kim et al., 2021a). We confirmed these findings in men in this study.

Previous microbiome studies based on the skin–gut axis have reported that various skin diseases are accompanied by variations in the gut microbiome (Salem et al., 2018; De Pessemier et al., 2021). Although significant differences in alpha and beta diversity indices could not be identified, we aimed to provide insights into gut–AGA through differential abundance of taxa between CON and AGA+ in Figure 4B. *Bifidobacterium*, which is much more abundant in healthy women, maintains intestinal homeostasis and is used as a probiotic in preventive medicine (Tojo et al., 2014; Sarkar and Mandal, 2016). Although generalization is difficult owing to the differences between men and women, *Lachnoclostridium*, *Butyricoccus*, and *Coprococcus* are beneficial bacteria that produce short-chain fatty acids that improve the intestinal environment through several local effects (Vital et al., 2014; Zhang et al., 2020; Nogal et al., 2021) and may act as key factors in clarifying the relationship between the gut microbiome and AGA.

We confirmed functional differences through variations in metagenomic pathways predicted by the bacterial communities. The metabolism of cofactors and vitamins category, which was enriched in the scalp microbiome of CON, is closely related to hair growth (Patel et al., 2017; Almohanna et al., 2019). Alpha lipoic acid acts as an antioxidant and blocks enzymes to reduce apoptosis of hair cells (Kim et al., 2014). Similarly, folate plays a role in inducing high proliferation of hair follicles by increasing the production of red blood cells (Mahmood, 2014). In a case study of 29 patients with AA, red blood cell folate concentrations of the scalp were significantly lower in the patients than in healthy controls (Almohanna et al., 2019). In the gut microbiome, the biosynthetic pathway of bile acid, which serves as an important mediator in the gut microbiome, was significantly enriched in healthy women. Primary bile acids produced in the liver are bioconverted into secondary bile acids by gut microbes (Staley et al., 2017), and secondary substances exert direct and indirect antimicrobial effects on pathogenic bacteria in the gut (Begley et al., 2005), thereby affecting the physiology of the host (Ridlon et al., 2014). Additionally, the bacterial invasion of the epithelial cell pathway was enriched in AGA+ in both sexes. Mucosal barrier dysfunction can lead to invasion of the intestinal mucosa by pathogenic bacteria, inducing excessive immunity of the host, which can result in intestinal inflammation (Okumura and Takeda, 2017, 2018). Although it was not possible to clearly determine intestinal dysbiosis in AGA+ in our study, its occurrence can be inferred through functional evaluation of bacteria, suggesting a new potential link between the gut microbiome and AGA.

Previous studies have suggested that studying the skin microbial network is more important than controlling and regulating specific bacteria to understand dandruff and to maintain younger skin (Kim et al., 2019). In our cohort, the scalp and gut microbiome were more complex and compact (higher density) in AGA+ with a higher value of network topological statistic coefficients including degree centrality, vertices, and edges. This may be because of the high moisture content in AGA+ causing colonization by non-inhabitant bacteria, which induce scalp microbial imbalance. Moreover, we confirmed more negative associations with AGA+ than with CON, suggesting increased negative interrelationships such as competition and predation. In particular, in the scalp microbiome, the increased bacterial diversity in AGA individuals compared with that in healthy controls can be understood in the same context as an increase in density and transitivity (clustering) in the network and an increase in the ratio of unique associations among overall associations. Also, increased appearance of non-inhabitant bacteria is associated with these features of network in AGA. Based on these results, AGA may be affected by multiple bacteria and caused by dysbiosis of the scalp microbiome, rather than by individual bacteria.

We acknowledge the following limitations related to our study. Our study provides preliminary evidence of an association between AGA and the microbiome. Further studies are needed to evaluate the role of bacteria in AGA. In addition, we identified bacteria based on amplicon sequencing and performed microbial functional evaluation using the prediction tool (PICRUSt2). Therefore, we could not experimentally confirm these results. Nevertheless, our data can be useful for further studies to elucidate the structure, function, and role of the microbiome in AGA. Our results can not only lead to the development of cosmetics and therapeutics using microorganisms and metabolites attributed to AGA but also provide a scientific basis from a microbiological perspective.

## Data availability statement

The data presented in the study are deposited in the SRA repository, accession number PRJNA891901 and PRJNA891926.

## Ethics statement

This study involving human participants was reviewed and approved by the Korean Public Institutional Review Board (IRB) of the Korea National Institute for Bioethics Policy (IRB: P01-202002–33-001).

## Author contributions

D-RJ: formal analysis, investigation, data curation, writing - original draft, and visualization. H-YY: conceptualization, methodology, investigation, resources, and writing - original draft. M-JK: methodology and investigation. VS: writing - review and editing. S-HP: investigation and resources. MJ: formal analysis. B-JP: project administration. J-HS: project administration and funding acquisition. All authors contributed to the article and approved the submitted version.

## Funding

This research was supported by a project to train professional personnel in biological materials by the Ministry of Environment, the Korea Basic Science Institute (National research Facilities and Equipment center) grant funded by the Ministry of Education (2021R1A6C101A416), and the Commercialization Promotion Agency for R&D Outcomes (COMPA) grant funded by the Korean Government (Ministry of Science and ICT)” (2022).

## Conflict of interest

H-YY, S-HP and B-JP were employed by Kolmar Korea Co., Ltd.

The remaining authors declare that the research was conducted in the absence of any commercial or financial relationships that could be construed as a potential conflict of interest.

## Publisher’s note

All claims expressed in this article are solely those of the authors and do not necessarily represent those of their affiliated organizations, or those of the publisher, the editors and the reviewers. Any product that may be evaluated in this article, or claim that may be made by its manufacturer, is not guaranteed or endorsed by the publisher.

## Footnotes

^1^ http://www.r-project.org/

^2^ https://github.com/vmikk/metagMisc
