# Supplementary material for: Comparative analysis of scalp and gut microbiome in androgenetic alopecia: A Korean cross-sectional study
Source: Front Microbiol. 2022 Dec 12;13:1076242. doi: 10.3389/fmicb.2022.1076242 (PMC9791053; doi:10.3389/fmicb.2022.1076242)
Supplement: Supplementary file 1 [file Data_Sheet_1.PDF]

Supplementary Material

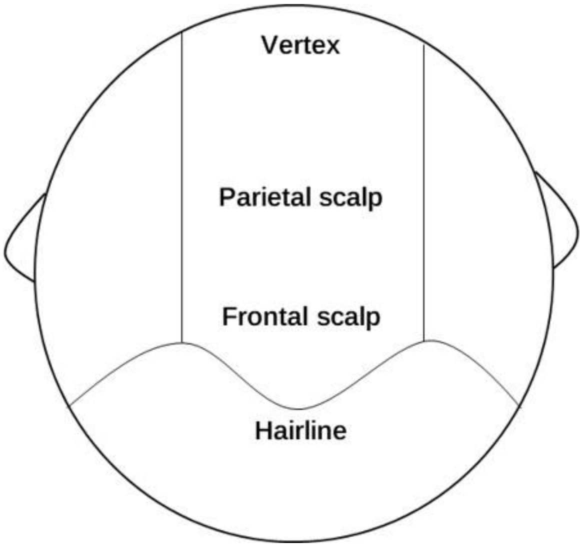

Supplementary Figure 1. Nomenclature for the scalp area.

(A)

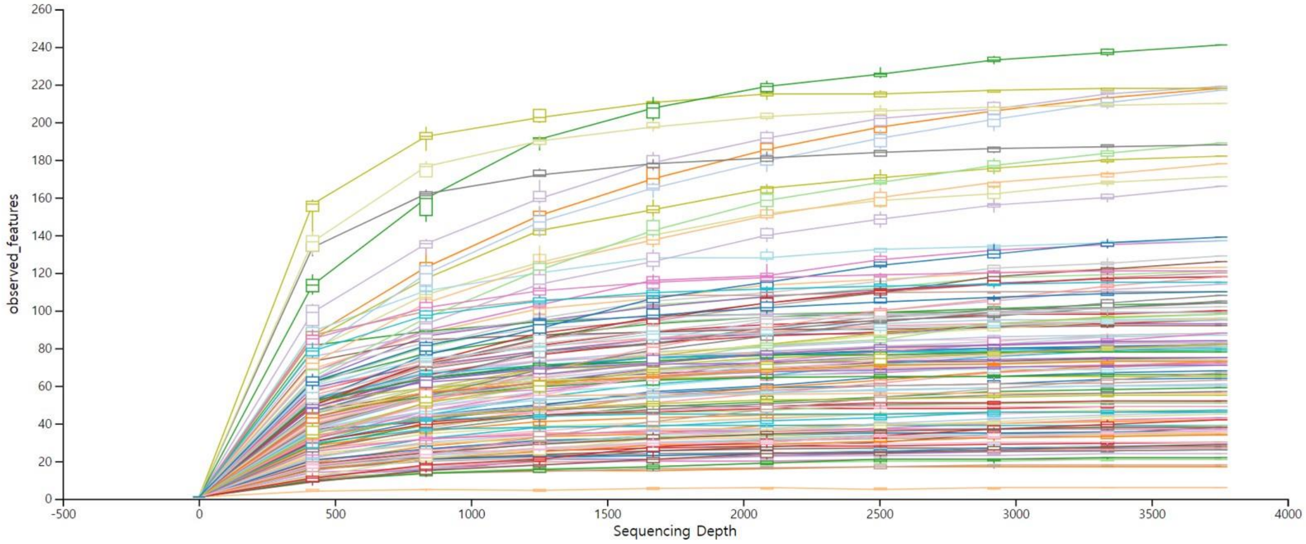

(B)

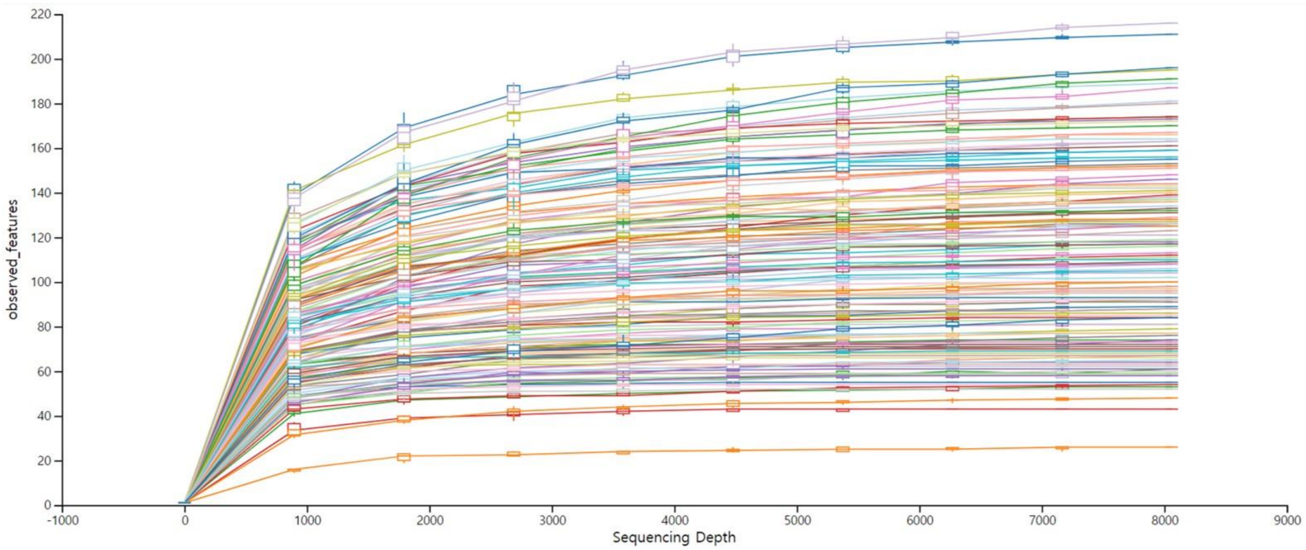

Supplementary Figure 2. Rarefaction curves of Illumina MiSeq sequencing. **(A)** Rarefaction curve of scalp bacterial sequencing with 3,756 of sequencing depth. **(B)** Rarefaction curve of fungal sequencing with 8,062 of sequencing depth.

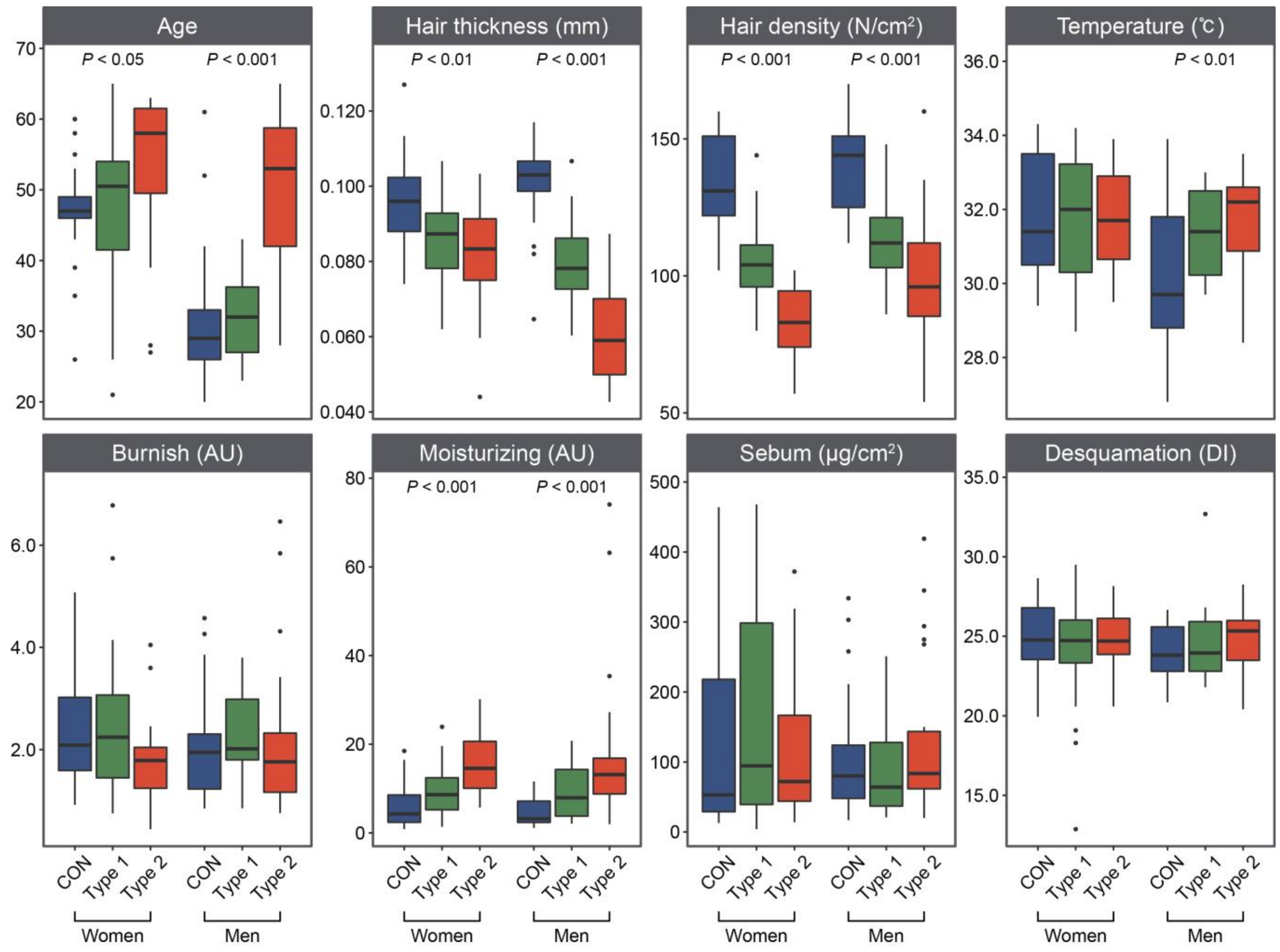

Supplementary Figure 3. Comparison of hair and scalp parameter measurement between healthy controls (CON), AGA type 1, and AGA type 2.

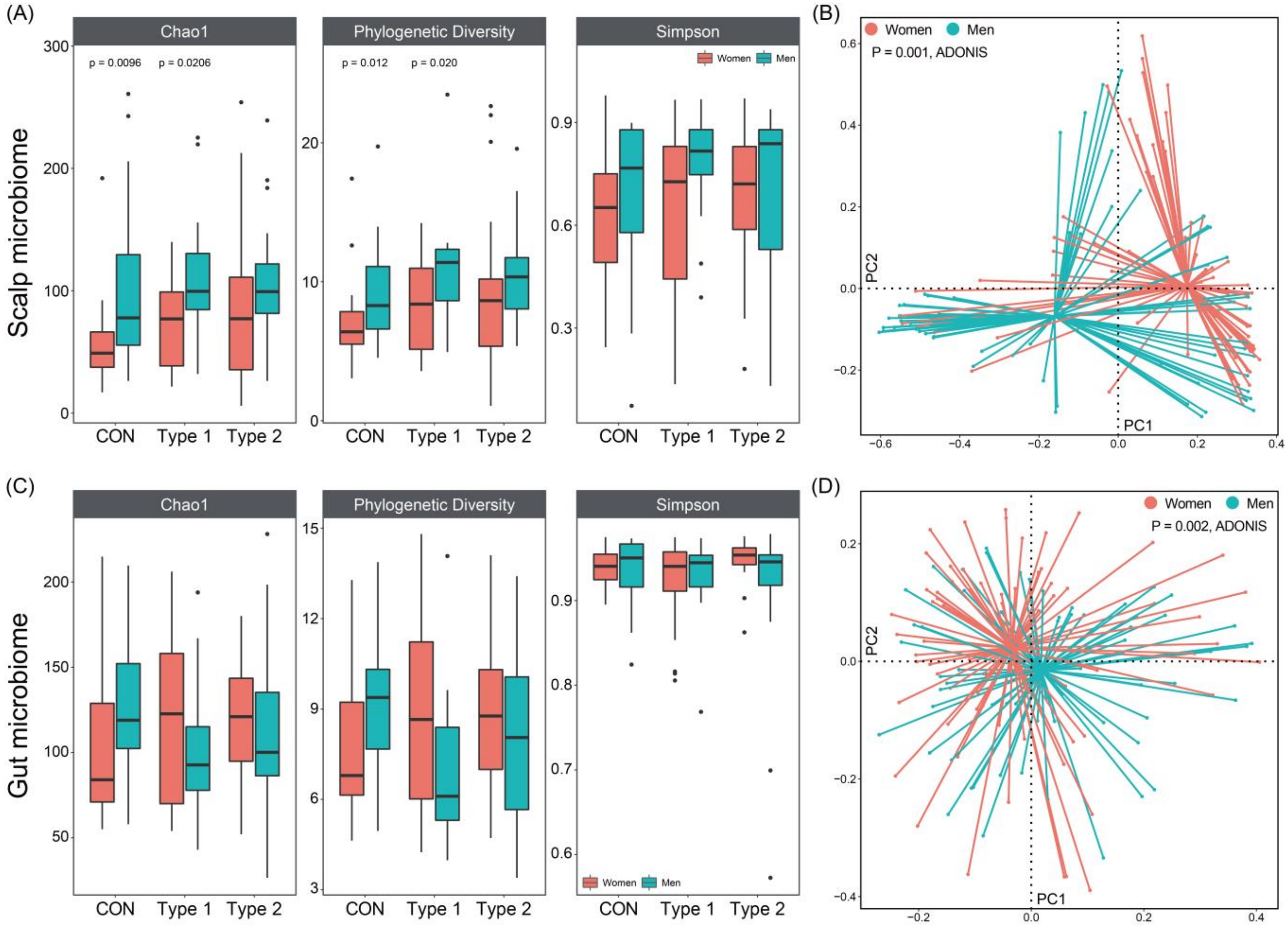

Supplementary Figure 4. The alpha and beta diversity of scalp microbiome according to sex. The comparison of the alpha diversity indices between women and men in the scalp **(A)** and gut **(C)** microbiome was evaluated by Wilcoxon rank-sum test. P values < 0.05 and 0.01 are indicated with * and **, respectively. Principal coordinates analysis plot (based on Bray–Curtis distances) in the scalp **(B)** and gut **(D)** microbiome.

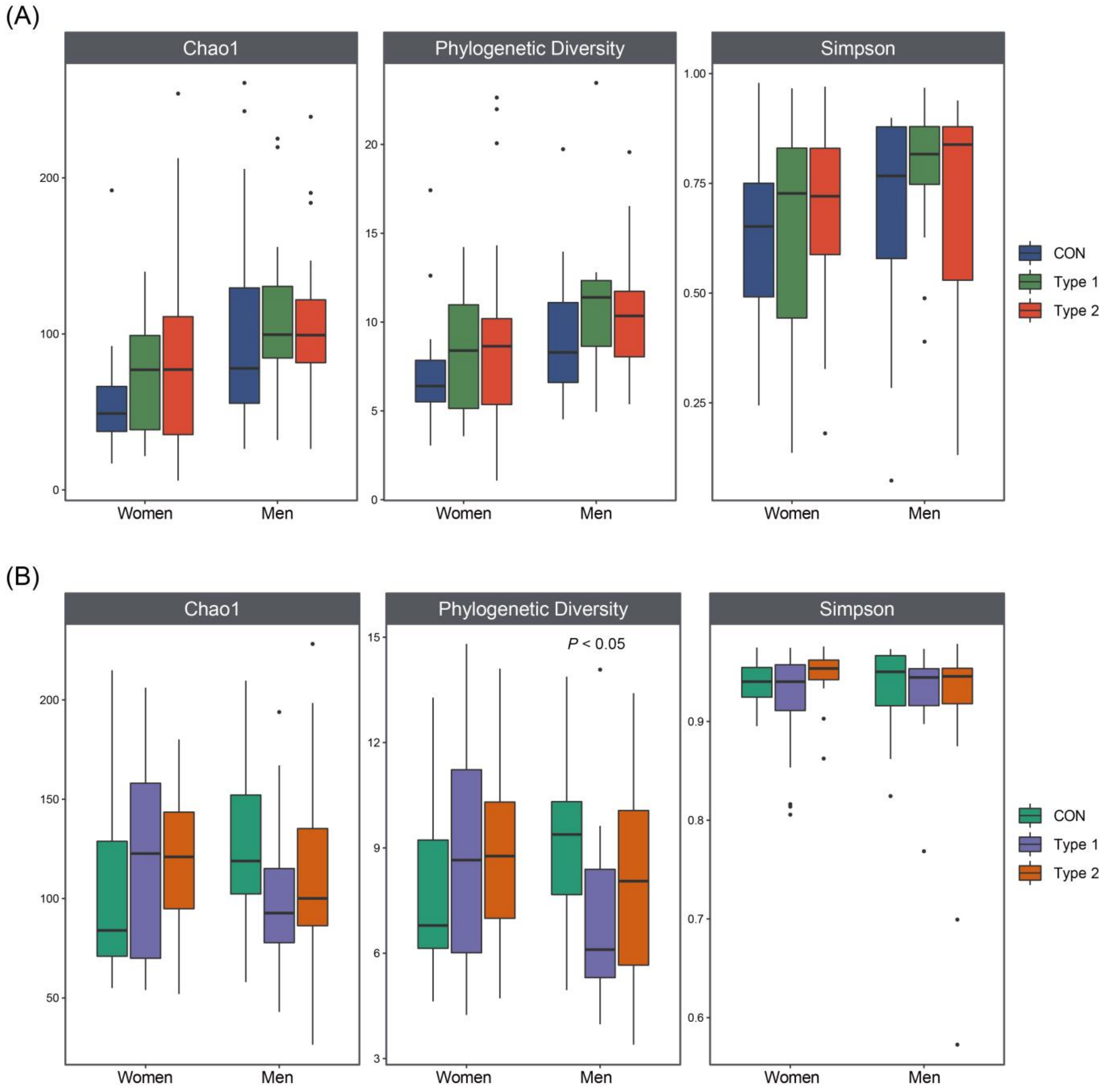

Supplementary Figure 5. Alpha diversity of scalp and gut microbiome according to the severity of AGA. The comparison of the alpha diversity indices between three groups was calculated by the Kruskal-Wallis test.

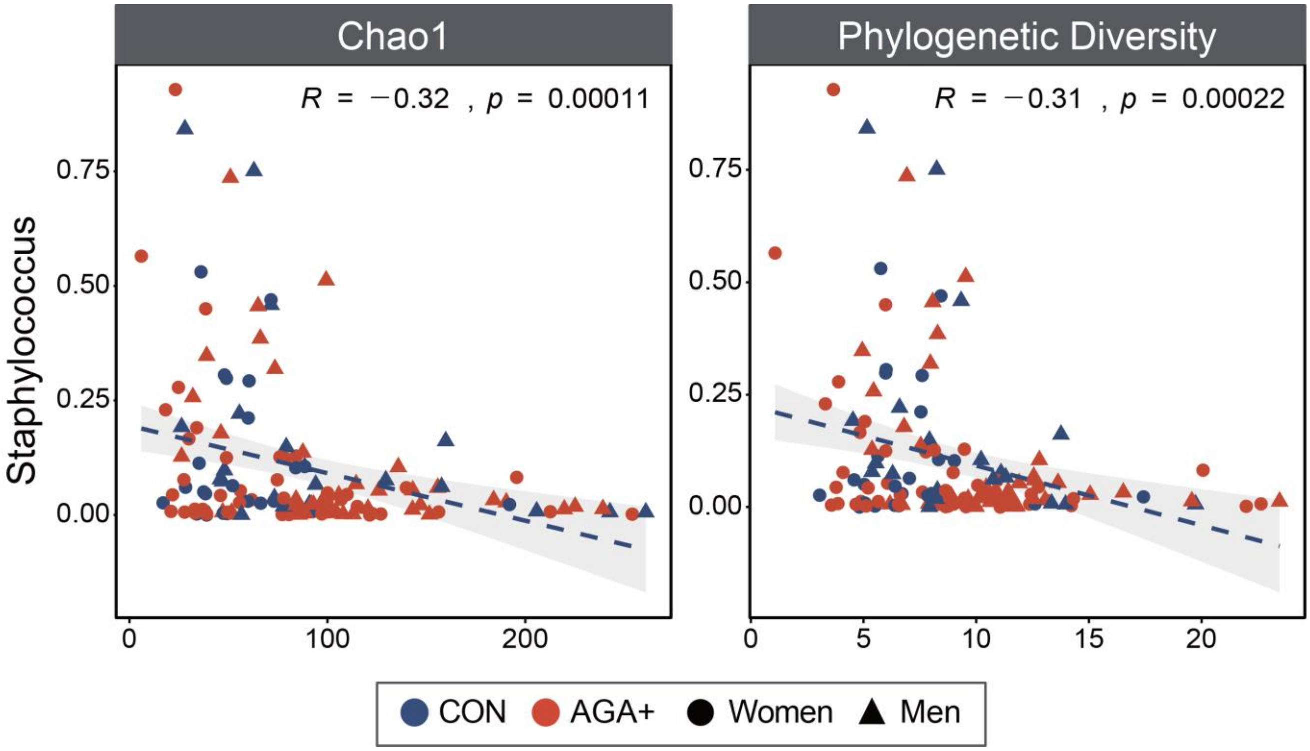

Supplementary Figure 6. Scatter plots with the correlation between *Staphylococcus* and alpha diversity indices of the scalp microbiome.

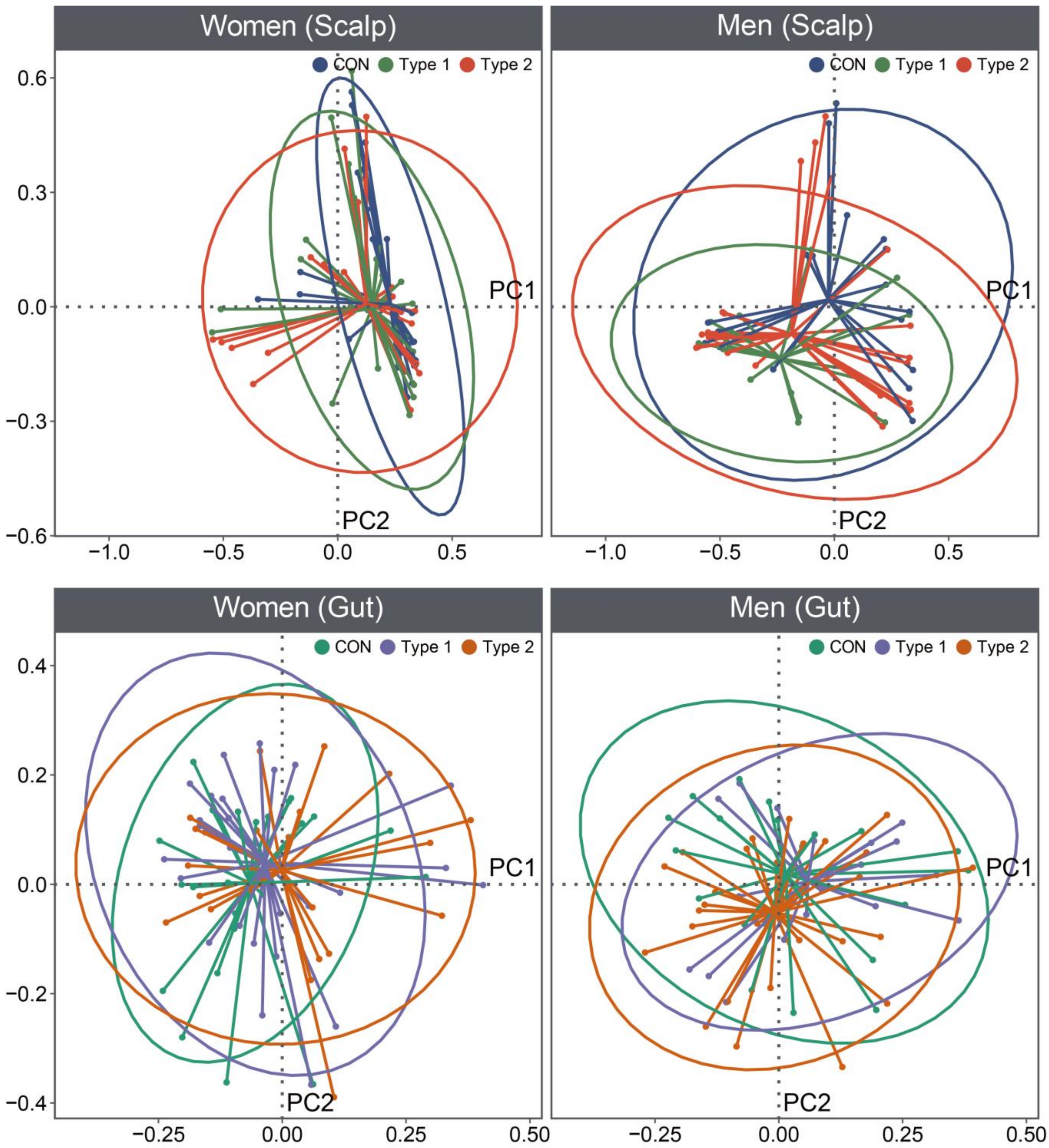

Supplementary Figure 7. Beta diversity of scalp and gut microbiome according to the severity of AGA.

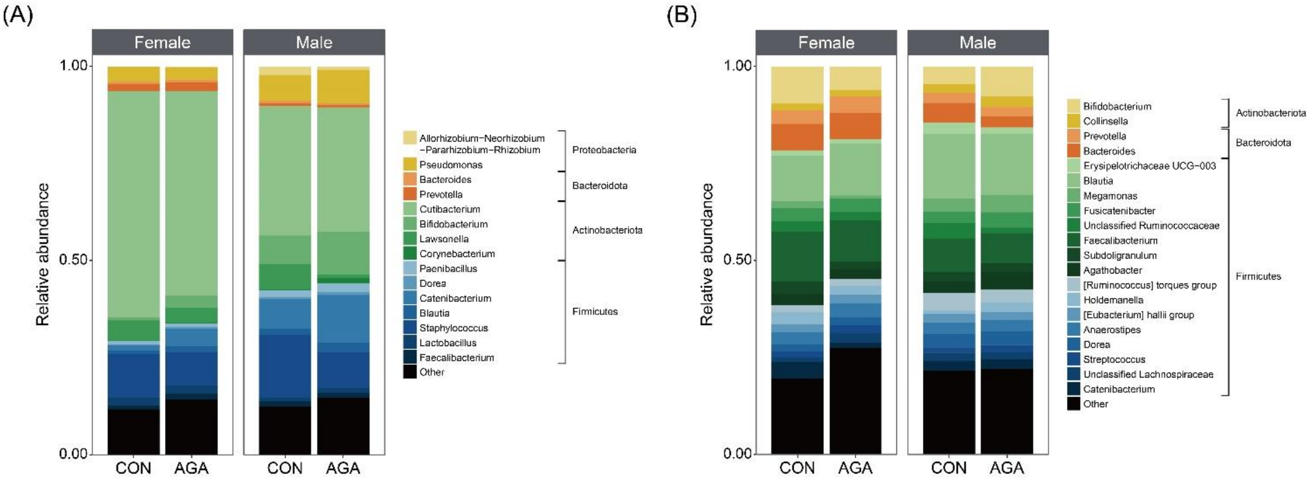

Supplementary Figure 8. Top 15 abundant genera in the scalp **(A)** and gut **(B)** microbiome.

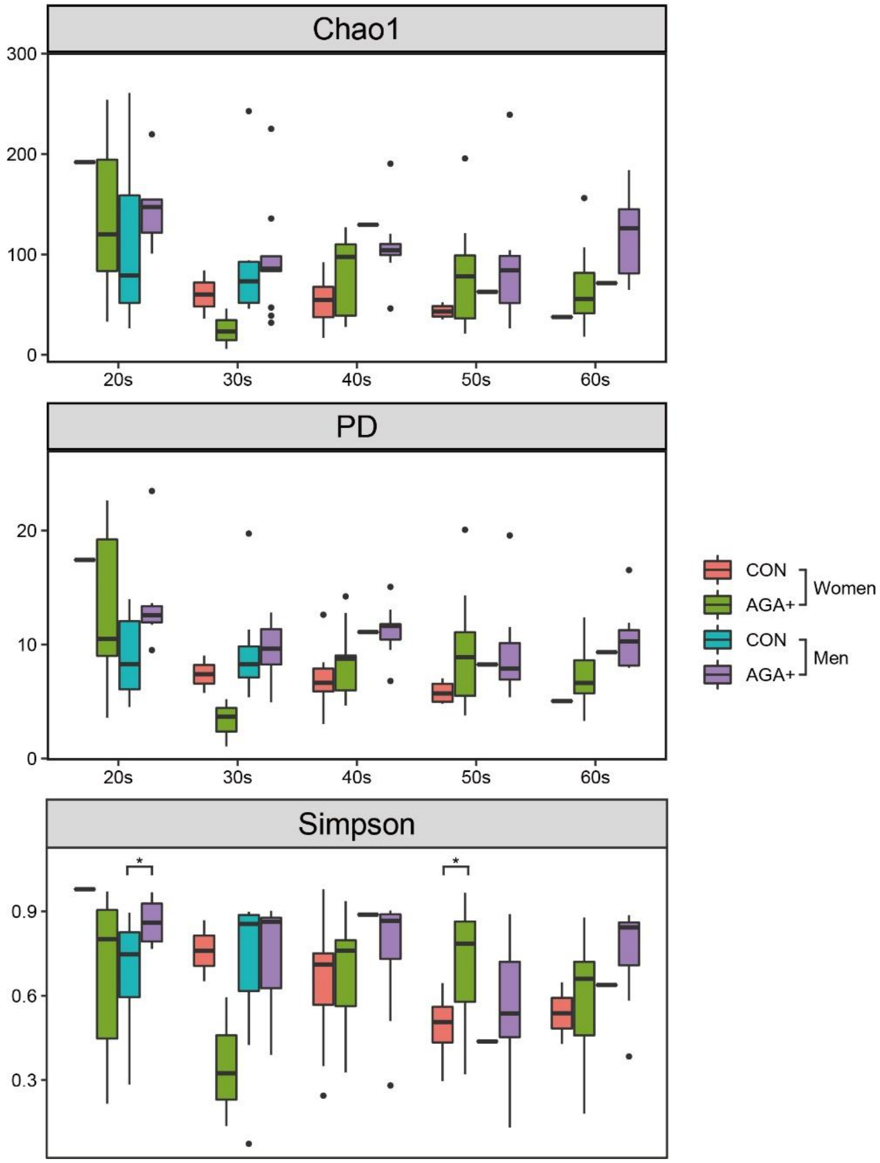

Supplementary Figure 9. Alpha diversity of scalp microbiome according to age and AGA. The comparison of the alpha diversity indices between three groups was calculated by the Wilcoxon rank-sum test. P value < 0.05 is indicated with *.
